# The prevalence of diagnosed chronic conditions and multimorbidity in Australia: A method for estimating population prevalence from general practice patient encounter data

**DOI:** 10.1371/journal.pone.0172935

**Published:** 2017-03-09

**Authors:** Christopher Harrison, Joan Henderson, Graeme Miller, Helena Britt

**Affiliations:** 1 Menzies Centre for Health Policy, Sydney School of Public Health, University of Sydney, Sydney, New South Wales, Australia; 2 Family Medicine Research Centre, Sydney School of Public Health, University of Sydney, Sydney, New South Wales, Australia; University of Oxford, UNITED KINGDOM

## Abstract

**Objectives:**

To estimate the prevalence of common chronic conditions and multimorbidity among patients at GP encounters and among people in the Australian population. To assess the extent to which use of each individual patient’s GP attendance over the previous year, instead of the average for their age-sex group, affects the precision of national population prevalence estimates of diagnosed chronic conditions.

**Design, setting and participants:**

A sub-study (between November 2012 and March 2016) of the Bettering the Evaluation and Care of Health program, a continuous national study of GP activity. Each of 1,449 GPs provided data for about 30 consecutive patients (total 43,501) indicating for each, number of GP attendances in previous year and all diagnosed chronic conditions, using their knowledge of the patient, patient self-report, and patient's health record.

**Results:**

Hypertension (26.5%) was the most prevalent diagnosed chronic condition among patients surveyed, followed by osteoarthritis (22.7%), hyperlipidaemia (16.6%), depression (16.3%), anxiety (11.9%), gastroesophageal reflux disease (GORD) (11.3%), chronic back pain (9.7%) and Type 2 diabetes (9.6%).

After adjustment, we estimated population prevalence of hypertension as 12.4%, 9.5% osteoarthritis, 8.2% hyperlipidaemia, 8.0% depression, 5.8% anxiety and 5.2% asthma. Estimates were significantly lower than those derived using the previous method.

About half (51.6%) the patients at GP encounters had two or more diagnosed chronic conditions and over one third (37.4%) had three or more. Population estimates were: 25.7% had two or more diagnosed chronic conditions and 15.8% had three or more.

**Conclusions:**

Of the three approaches we have tested to date, this study provides the most accurate method for estimation of population prevalence of chronic conditions using the GP as an expert interviewer, by adjusting for each patient’s reported attendance.

## Introduction

Australia has a universal medical insurance scheme called Medicare which (fully or partially) covers the individuals cost of visits to general practitioners (GPs). GPs are paid on a fee-for-service basis. There is no patient registration, patients being free to visit any number practices and GPs as they choose. In any single year around 85% of Australians see a GP at least once[1] with GPs providing the bulk of primary care and acting as gate-keepers to government-subsidised health care from other health professionals.

Like all OECD countries, Australia’s population is ageing[2,3]. It is expected this will increase the prevalence of diagnosed chronic conditions[4,5], of multimorbidity[6–8], and demand on the health care system[7,9,10]. In response, the Australian federal government recently announced a “Health Care Home” (Patient Centred Medical Home) initiative whereby patients with chronic and complex conditions voluntarily enrol at a general practice[11]. This plan will include a “bundled payment” (partial capitation) to the practice for each patient enrolled. While initial reports implied that patients with multiple chronic conditions would be targeted by the initiative, recent announcements suggest that patient eligibility will be determined by their risk of hospital admission[12]. However, hospital admission risk may not accurately predict use of general practice services, yet this will be required to calculate fair compensation to GPs under the partial capitation model of the initiative. Preliminary results have shown that multimorbidity is a strong predictor of primary care resource use[13]. Therefore to cost this initiative, the prevalence of chronic conditions and multimorbidity needs to be measured accurately.

Large population health surveys that rely on respondent self-report are commonly used to measure the prevalence of chronic conditions[14–16]. One such study is the National Health Survey (NHS)[17], one of Australia's largest health surveys, undertaken by the Australian Bureau of Statistics every three to six years since 1989. The most recent (2014–15) surveyed 19,259 people from 14,723 households, and while it used some measured data (such as respondent’s blood pressure, height, weight and waist circumference) it still relied on respondent self-report for measurement of the prevalence of chronic conditions.[17] This is despite concerns about the accuracy of self-reported health information[18–22].

Due to these concerns, review of health records (paper and/or electronic) is often assumed to be a more accurate way of estimating prevalence of chronic conditions. However, this approach has its own issues with the stored information sometimes being inaccurate and often incomplete[23–25]. There are also concerns around obtaining patient consent to use their data, with many patients not being informed[26].

The BEACH (Bettering the Evaluation And Care of Health) program was a study of GP clinical activity in Australia[1]. Sub-studies of the BEACH program allowed us to investigate aspects of health and health care delivery, free of the limitations of health record audits and patient self-report. The sub-studies utilised the GP as an expert interviewer and informant, drawing on the patient's knowledge, their knowledge of the patient, and the patient's health record.

A study conducted in 2005 showed that sub-studies embedded within the national BEACH program could provide timely, accurate prevalence estimates of common chronic conditions in Australia[4].

In 2008–09, we conducted another sub-study which built on our earlier methods by expanding the study's scope to include all chronic conditions (rather than a selection of common chronic conditions) and by improving the methods of dealing with non-attenders when estimating population prevalence[5].

However, in the earlier studies we were not able to adjust for high and low attenders to general practice within each age-sex group of patients. This meant that our national estimates may have been inflated if, within a specific age-sex group, people with more diagnosed chronic conditions attend more often than people without chronic conditions. This may be true as our 2008–09 study estimated that 32.6% of the population had two or more diagnosed chronic conditions, a significantly higher proportion than that of the 2014–15 NHS (23.0%)[17].

Since the 2008–09 study, we introduced an additional question asking how many times the patient had seen a GP in previous 12 months (including today’s visit). This will allow adjustment for attendance for each individual patient and overcomes the major limitation of the 2008–09 study.

If it is decided that the compensation paid to GPs for each patient enrolled in the health care home initiative is based on the patient’s multimorbidity load, the way multimorbidity is measured will also need to be decided. The most common way of measuring multimorbidity is a simple count of the number of diagnosed chronic conditions within a patient[7,27]. Alternatively, it has been suggested that it is not the number of individual chronic conditions that is important, but the number of body systems affected by these chronic conditions[8,27].

The aims of this study were to:

1. estimate the prevalence of common chronic conditions among patients at GP encounters and among people in the Australian population.
2. assess the extent to which use of each individual patient’s reported GP attendance over the previous year, instead of the average for their age-sex group, affects the precision of national population prevalence estimates of diagnosed chronic conditions.
3. estimate the prevalence of multimorbidity among patients at GP encounters and among people in the Australian population.

## Method

This study was undertaken as a sub-study of the BEACH program. BEACH was a continuous, national cross-sectional study of general practice activity in Australia operating from April 1998–March 2016 inclusive. Its methods are described in detail elsewhere.[1] In summary, each year an ever-changing, random sample of about 1,000 GPs each recorded information about encounters with 100 consecutive consenting patients, on structured paper forms.

In BEACH sub-studies, the GP recorded information additional to the encounter data, in discussion with the patient. In this sub-study, 1,800 participating GPs were each asked to record all diagnosed chronic conditions present in each of 30 consecutive patients within their 100 BEACH encounter forms over twelve five-week recording periods between 27th November 2012 and 28th March 2016.

GPs were instructed to ‘‘Use your own knowledge, patient knowledge and health records as you see fit, in order to answer these questions”. GPs were first asked, “Approx. how many times has this patient seen any GP in the past 12 months? (Including today)”. They were then asked ‘‘Does the patient have any chronic diseases/problems?”. If ‘No’, the GP ended the questions for that patient. If ‘Yes’, the GP indicated all the diagnosed chronic conditions for that patient. Tick boxes were provided for common chronic conditions and additional blank spaces were provided to allow free text recording of other unlisted chronic condition.

Chronic conditions listed were primarily those that were included in the previous prevalence study[5] based on those most frequently managed in Australian general practice[1]. Chronic conditions were classified according to the International Classification of Primary Care (Version 2) (ICPC-2)[28].

Examples of the instruction sheet and the recording form provided to the GP are attached in S2 and S3 Files. The final question (which was not analysed for this paper) varied over the sub-studies, however the variables analysed in this paper were asked consistently across the sub-studies.

### Data analysis

In previous studies[4,5] we found that patients for whom no response was recorded for the chronic condition question were similar in terms of age and problems managed at their encounters, to patients for whom the “no chronic conditions” option was recorded. Based on these similarities, we assumed that some GPs were leaving this question blank for patients who had no diagnosed chronic conditions. To account for this, patients with missing chronic condition data were counted as having “No chronic conditions” to ensure we did not overestimate the prevalence of chronic conditions. We then examined these same patient's encounter record to see whether any chronic conditions (as defined by O’Halloran et al[29]) were managed at their encounter. If chronic conditions were managed at the encounter, they were no longer considered to have “No chronic conditions” and those chronic conditions managed at their encounter were assigned to the patient in the sub-study. If in the current study we find that patients with missing chronic condition data were similar to those who had the “No chronic conditions” option ticked, we will follow the steps described above from previous studies.

When the number of GP visits in the previous year was not recorded (missing data), the average number of visits for a patient in the same 10 year age group, the same sex and the same number of diagnosed chronic conditions (0,1,2,3+ chronic conditions) was assigned.

Multimorbidity was defined as the “co-occurrence of two or more chronic conditions within one person without defining an index chronic condition” and complex multimorbidity as the “co-occurrence of three or more chronic conditions affecting three or more different body systems within one person without defining an index chronic condition”[27]. The chapters of ICPC-2 were used to represent the different body systems. A patient with complex multimorbidity had at least one diagnosed chronic condition in each of three or more different ICPC-2 chapters. Body systems were counted only once per patient, even if they had multiple chronic conditions classified to that body system.

The proportion of patients with morbidity X in the unweighted sample can be interpreted as the prevalence of that condition among patients found in GP waiting rooms or at GP encounters. We compared the prevalence of common chronic conditions at GP encounters with two earlier studies (Knox et al.[4] & Harrison et al.[5]) that used the same method. The only differences between the studies were that Knox et al. used a limited number of conditions and the conditions listed in Harrison et al[5] were listed in a different order.

As patients were sampled at GP consultations, the likelihood of being sampled is dependent on visit frequency. Therefore frequent attenders (such as older patients who may have more health problems) were more likely to be sampled than infrequent attenders.

In Harrison et al.[5], to estimate national prevalence, we weighted the data to match the age–sex distribution of the Australian population. We assumed that people who did not attend a GP that year had no diagnosed chronic conditions. After the above weighting we multiplied the outcome (condition count) for each patient, by the proportion of their age-sex group who saw a GP at least once that year (data supplied by the Australian Government Department of Health). This accounted for those who did not see a GP that year. However, this method did not account for high attenders within specific age-sex groups.

In the current study, we were able to adjust for high or low attenders by weighting each patient’s data by the number of times they reported seeing a GP in the previous year, with high attenders being weighted down and low attenders being weighted up. We then followed the previous method using the weighted data instead of the raw data. Table 1 demonstrates how the weightings were calculated for each of the two methods using two example patients.

**Table 1:** New and previous methods to weight “encounter” data to reflect “population” prevalence.

	Example 1: Male patient aged 10–14 years		Example 2: Female patient aged 80–84 years	
	Old Method	New Method	Old Method	New Method
Reported number of GP visits in previous year (A)	---	8	---	6
Average number of GP visits for total sample(B)	---	4.54	---	4.54
C = B/A (Weight to adjust for attendance)	1	0.57	1	0.76
Proportion of the Australian population (D)	3.10%	3.10%	1.09%	1.09%
Proportion of sample that was in the selected age-sex group (after weighting in the New method) (E)	1.18%	2.03%	3.15	1.47%
F = D/E (National weight)	2.63	1.53	0.35	0.74
G = Proportion of age-sex group that saw a GP at least once that year	74.85%	74.85%	96.53%	96.53%
Final adjustment of outcome (or numerator) to estimate national prevalence = C*F*G	1.97	0.65	0.34	0.54
Denominator for national estimates (for both patients with and without condition) = C*F	2.63	0.87	0.35	0.56

To test the effect of this new method on our estimates, we weighted the current data using both methods. We compared these national prevalence estimates with those of the previous study. If it is true that within an age-sex group, patients with chronic conditions attend more often than those without chronic conditions, then the prevalence estimates resulting from the new method should produce lower estimates than those produced by the previous method.

BEACH sub-studies have a single stage cluster design, with each GP having 30 patients clustered around them. Survey procedures (in SAS 9.3) were used to account for the effect of this clustering. Significant differences were determined by non-overlapping 95% confidence intervals (CIs). This is a more conservative estimate of difference than the usual p<0.05[30].

### Ethics statement

During the data collection period for this study the BEACH program was approved by the Human Research Ethics Committee of the University of Sydney (Reference number 2012/130). Our method involved the collection of data from unidentifiable, consenting patients. In the research kit, a patient information card was supplied and GPs were instructed to show this to patients in order to obtain informed consent (an example shown in Britt et al.[1]). If the patient chose not to participate, their encounter details were not recorded. GPs were instructed to note the patient’s consent in the patient’s record, but were not asked to provide written consent to the research body, to preserve patient anonymity. These methods comply with the Ethics requirements for the BEACH program.

## Results

Of the 1,800 GPs recruited, 1,449 GPs (80.5%) returned completed recording forms. Of the 43,501 patients in this sample, 41,722 (95.9%) reported the number of times they had seen a GP in the previous year and 42,185 (97.0%) responded to the chronic condition questions. The 1,316 patients with missing chronic condition data were examined and found to be similar to those patients with no chronic conditions, with both groups being younger on average than the total sample. Further, the most frequently managed problems at their encounters were acute, whereas in the total sample the most frequently managed conditions were chronic. Of these 1,316 patients, 323 (24.5%) had one or more chronic conditions managed at the encounter and were included as having these conditions while the remaining 993 (75.5%) were added to the no chronic conditions group (results not tabled).

On average, patients in the sample had seen a GP 9.66 times in the previous 12 months. After adjusting for this attendance, we estimated that all people who had seen a GP at least once in the previous 12 months, visited a GP 4.54 times on average.

Overall, the age-sex distribution of the sample was similar (range 0.80–1.14) to that of patients at all Medicare or Department of Veteran Affairs (DVA) claimed GP consultations, with the exception of patients aged less than 15 years (80–83% of expected) (Table 2). After adjusting for each patient’s attendance over the previous year, the age-sex distribution of the weighted sample was similar to that of all patients who had claimed at least one Medicare GP item of service within the previous year, with the exception of female patients aged 75 years and over (23% more than expected).

**Table 2:** Age-sex distribution of the sample.

Patient Age/Sex	Number in sample	Percent of sample (95% CI)	Percent of Australian general practice service claims*	Precision ratio	Percent of sample after adjusting for attendance	Percent of the Australian general practice population@	Precision ratio
**Male**							
<15 years	2,369	5.5% (5.2–5.8)	6.9%	0.80	8.1% (7.7–8.6)	9.4%	0.86
15–24 years	1,246	2.9% (2.7–3.1)	3.2%	0.91	5.0% (4.7–5.4)	5.5%	0.92
25–44 years	3,210	7.5% (7.1–7.9)	8.8%	0.85	11.0% (10.4–11.6)	12.3%	0.89
45–64 years	4,735	11.0% (10.6–11.4)	11.2%	0.99	11.6% (11.1–12.2)	12.3%	0.95
65–74 years	2,756	6.4% (6.1–6.7)	6.0%	1.07	4.8% (4.5–5.1)	4.5%	1.06
75+ years	3,011	7.0% (6.6–7.4)	6.8%	1.03	3.5% (3.3–3.8)	3.3%	1.09
**Female**							
<15 years	2,236	5.2% (4.9–5.5)	6.3%	0.83	7.7% (7.2–8.2)	8.9%	0.87
15–24 years	2,159	5.0% (4.7–5.3)	5.5%	0.91	6.6% (6.1–7.0)	6.3%	1.04
25–44 years	6,057	14.1% (13.6–14.6)	14.4%	0.98	15.7% (15.0–16.3)	14.8%	1.06
45–64 years	6,927	16.1% (15.7–16.6)	14.6%	1.10	15.0% (14.4–15.6)	13.6%	1.11
65–74 years	3,593	8.4% (8.0–8.7)	6.8%	1.22	5.7% (5.4–6.0)	4.9%	1.18
75+ years	4,605	10.7% (10.2–11.3)	9.4%	1.14	5.2% (4.9–5.5)	4.2%	1.23

There were 492 patients who had either/both age and/or sex missing.

*All general practice Medicare Benefits Schedule (MBS) items claimed GPs in 2014–15 and all Department of Veteran Affairs GP claims in 2012–13 (Most recent year available). MBS data supplied by the Medicare Information Analysis Section and Department of Veteran Affairs data was supplied by the Department of Veteran Affairs.

@Distribution of all patients who had at least one MBS GP service item claimed in 2014–15

### Sample prevalence of individual chronic conditions

The circulatory system was the body system most commonly affected by a chronic condition, with nearly a third (32.4%) of patients at GP encounters having at least one diagnosed circulatory chronic condition (Table 3). The musculoskeletal system and connective tissue (32.1%); and the endocrine, nutritional and metabolic disease system (30.7%) were also commonly affected by at least one diagnosed chronic condition. About one quarter (26.7%) of patients at GP encounters had a diagnosed psychological problem.

**Table 3:** Prevalence of common diagnosed chronic conditions among patients at GP encounters across three studies.

	Knox et al. estimates (2005, n = 9,156)	Harrison et al. (2008–09, n = 8,707)	Current estimates (2012–16, n = 43,501)
**Circulatory**	**30.0% (28.1–31.7)**	**31.3% (29.4–33.1)**	**32.4% (31.5–33.4)**
Hypertension	23.3% (21.8–24.9)	26.6% (24.9–33.1)	26.5% (25.6–27.3)
Ischaemic Heart Disease	9.5% (8.5–10.5)	8.7% (7.7–9.8)	7.8% (7.4–8.2)
Cerebrovascular Accident	3.7% (3.0–4.5)	2.9% (2.3–3.5)	2.6% (2.4–2.8)
Congestive Heart Failure	3.2% (2.7–3.7)	2.9% (2.4–3.4)	2.6% (2.4–2.8)
Peripheral Vascular Disease	2.0% (1.5–2.5)	N/A	1.8% (1.7–2.0)
**Musculoskeletal system and connective tissue**	**N/A**	**26.4 (24.6–28.2)**	**32.1% (31.1–33.0)**
Any Arthritis	22.8% (21.1–24.5)	19.7% (18.1–21.4)	25.0% (24.1–25.9)
Rheumatoid	1.0% (0.8–1.2)	1.0% (0.7–1.2)	1.3% (1.2–1.5)
Osteoarthritis	20.0% (18.3–21.6)	17.8% (16.2–19.4)	22.7% (21.8–23.6)
Other and unknown	N/A	2.0% (1.7–2.4)	2.0% (1.9–2.2)
Chronic Back Pain	10.1% (9.0–11.1)	6.4% (5.5–7.2)	9.7% (9.2–10.2)
Osteoporosis	N/A	4.8% (4.2–5.5)	5.8% (5.4–6.1)
**Endocrine, nutritional and metabolic diseases**	**N/A**	**30.8% (29.0–32.6)**	**30.7% (29.9–31.6)**
Hyperlipidaemia	15.9% (14.7–17.2)	18.5% (17.0–20.0)	16.6% (15.9–17.3)
Diabetes all	8.3% (7.5–9.0)	9.2% (8.3–10.1)	10.4% (10.0–10.8)
Type 1	0.6% (0.4–0.8)	0.9% (0.6–1.2)	0.9% (0.8–1.0)
Type 2	7.2% (6.5–7.9)	8.3% (7.5–9.1)	9.6% (9.2–10.0)
**Psychological Problems**	**24.8% (23.2–26.3)**	**22.1% (20.6–23.7)**	**26.7% (25.9–27.5)**
Depression	14.2% (13.0–15.4)	13.7% (12.6–14.7)	16.3% (15.8–16.9)
Anxiety	10.7% (9.6–11.8)	8.3% (7.3–9.4)	11.9% (11.4–12.4)
Insomnia	5.5% (4.6–6.4)	N/A	3.7% (3.4–4.0)
**Digestive**	**N/A**	**14.6% (13.4–15.8)**	**15.1% (14.5–15.7)**
GORD	13.1% (11.9–14.4)	11.6% (10.5–12.6)	11.3% (10.7–11.8)
**Respiratory Disease**	**N/A**	**13.7% (12.6–14.7)**	**14.6% (14.1–15.1)**
Asthma	10.7% (9.8–11.6)	9.5% (8.7–10.3)	8.3% (8.0–8.7)
COAD/COPD	3.6% (3.1–4.2)	4.1% (3.4–4.7)	4.5% (4.2–4.7)
**Malignant Neoplasms**	**3.1% (2.6–3.6)**	**5.0% (4.4–5.7)**	**6.2% (5.9–6.5)**

Note: GORD = gastro oesophageal reflux disease, COAD/COPD chronic obstructive airways disease/chronic obstructive pulmonary disease

N/A: Result not available due to chronic condition not being measured

Hypertension (26.5%) was the most prevalent individual diagnosed chronic condition, followed by osteoarthritis (22.7%), hyperlipidaemia (16.6%), depression (16.3%), anxiety (11.9%), gastroesophageal reflux disease (GORD) (11.3%), chronic back pain (9.7%) and Type 2 diabetes (9.6%).

The prevalence estimates for diagnosed ischaemic heart disease, cerebrovascular accidents, GORD and asthma among patients at GP encounters were significantly lower than the 2005 study estimates. Conversely, the prevalence estimates of diagnosed hypertension, osteoarthritis, Type 2 diabetes, depression and malignant neoplasms were each significantly higher than in the 2005 study.

### Population prevalence of individual conditions

After adjustment, we estimated that 16.0% of people in the population had at least one endocrine, nutritional and metabolic disease, 15.0% had at least one circulatory condition and 14.4% had at least one musculoskeletal system and connective tissue chronic condition (Table 4).

**Table 4:** Population prevalence of common diagnosed chronic conditions and multimorbidity.

	Harrison et al. (2008–09, n = 8,707) (95% CIs)	Current using previous method (2012–15, n = 43,501) (95% CIs)	Current using revised method (2012–15, n = 43,501) (95% CIs)	National Health Survey (2014–15, n = 19,259) (95% CIs)
**Circulatory**	**19.6% (18.3–20.9)**	**18.5% (17.9–19.2)**	**15.0% (14.3–15.6)**	**18.3% (17.7–18.9)**
Hypertension	16.6% (15.4–17.8)	15.1% (14.5–15.7)	12.4% (11.8–12.9)	11.3% (10.8–11.8)
Ischaemic Heart Disease	5.0% (4.4–5.6)	4.0% (3.8–4.2)	2.9% (2.7–3.1)	**
Cerebrovascular Accident	1.5% (1.2–1.8)	1.3% (1.2–1.4)	0.9% (0.8–1.0)	0.8% (0.6–1.0)
Congestive Heart Failure	1.5% (1.2–1.8)	1.2% (1.1–1.3)	0.8% (0.7–0.8)	0.5% (0.4–0.6)
Peripheral Vascular Disease	N/A	0.9% (0.8–1.0)	0.6% (0.5–0.6)	**
**Musculoskeletal system and connective tissue**	**16.7% (15.5–18.0)**	**19.2% (18.5–19.9)**	**14.4% (13.8–15.1)**	**
Any Arthritis	11.9% (10.8–12.9)	13.9% (13.3–14.5)	10.7% (10.1–11.2)	15.3% (14.8–15.8)
Rheumatoid	0.6% (0.4–0.7)	0.8% (0.7–0.9)	0.6% (0.5–0.6)	1.8% (1.6–2.0)
Osteoarthritis	10.4% (9.4–11.4)	12.3% (11.7–12.8)	9.5% (9.0–10.0)	9.0% (8.6–9.4)
Other and unknown	1.5% (1.2–1.7)	1.4% (1.3–1.6)	1.0% (0.9–1.2)	5.3% (4.9–5.7)
Chronic Back Pain	4.4% (3.8–5.0)	6.5% (6.2–6.9)	4.1% (3.8–4.3)	**
Osteoporosis	2.4% (2.1–2.8)	2.6% (2.4–2.8)	2.1% (1.9–2.2)	3.5% (3.2–3.8)
**Endocrine, nutritional and metabolic diseases**	**21.3% (19.9–22.6)**	**20.0% (19.4–20.6)**	**16.0% (15.3–16.6)**	**13.8% (13.3–14.3)**
Hyperlipidaemia	12.3% (11.3–13.4)	10.0% (9.5–10.4)	8.2% (7.7–8.6)	7.1% (6.7–7.5)
Diabetes all	6.1% (5.5–6.7)	6.4% (6.2–6.7)	4.6% (4.4–4.9)	5.1% (4.8–5.4)
Type 1	0.7% (0.5–0.9)	0.7% (0.6–0.8)	0.5% (0.4–0.6)	0.7% (0.5–0.9)
Type 2	5.5% (4.9–6.0)	5.8% (5.5–6.0)	4.2% (3.9–4.4)	4.4% (4.1–4.7)
**Psychological Problems**	**16.6% (15.3–17.8)**	**20.5% (19.8–21.2)**	**13.7% (13.1–14.2)**	**17.5% (16.8–18.2)**
Depression	10.0% (9.2–10.8)	12.5% (12.0–13.0)	8.0% (7.6–8.4)	8.9% (8.4–9.4)
Anxiety	6.2% (5.4–7.0)	9.3% (8.9–9.8)	5.8% (5.5–6.2)	**
Insomnia	N/A	2.4% (2.2–2.6)	1.5% (1.3–1.6)	N/A
**Digestive**	**9.6% (8.8–10.4)**	**9.9% (9.4–10.3)**	**7.1% (6.7–7.5)**	**6.2% (5.8–6.6)**
GORD	7.5% (6.8–8.2)	6.9% (6.5–7.2)	4.9% (4.6–5.2)	N/A
**Respiratory Disease**	**10.5% (9.7–11.4)**	**11.1% (10.7–11.5)**	**7.9% (7.6–8.3)**	**
Asthma	7.8% (7.1–8.5)	7.1% (6.8–7.4)	5.2% (4.9–5.5)	10.8% (10.2–11.4)
COPD	2.5% (2.1–2.9)	2.4% (2.2–2.6)	1.6% (1.5–1.7)	2.6% (2.3–2.9)
**Malignant Neoplasms**	**3.1% (2.7–3.6)**	**3.4% (3.2–3.6)**	**2.8% (2.6–3.0)**	**1.6% (1.4–1.8)**

Note: GORD = gastro oesophageal reflux disease, COPD chronic obstructive pulmonary disease

** Inclusions used by NHS too different for reasonable comparison, N/A Results not available

Hypertension was the most prevalent condition (12.4% of the population) followed by osteoarthritis (9.5%), hyperlipidaemia (8.2%), depression (8.0%), anxiety (5.8%), asthma (5.2%), GORD (4.9%), Type 2 diabetes (4.2%) and chronic back pain (4.1%).

Almost all the population prevalence estimates using the new ‘revised’ method were significantly lower than the 2008–09 prevalence estimates. However, when the current study’s data were analysed using the older method (which adjusted for age-sex group attendance averages rather than individual patient’s attendance) the prevalence estimates did not significantly differ from those found in the previous study. The population prevalence estimates produced using the new method were significantly lower (between 18% lower for dementia to 40% lower for insomnia) than those derived using the previous method when using the same data.

Compared with the 2014–15 NHS estimates, our prevalence estimates were significantly higher for circulatory conditions (including congestive heart failure), endocrine, nutritional and metabolic disease (including hyperlipidaemia), gastrointestinal conditions and malignant neoplasms. Our prevalence estimates were significantly lower for total arthritis, rheumatoid arthritis, other arthritis, osteoporosis, asthma and chronic obstructive pulmonary disease than the 2014–15 NHS estimates.

### Prevalence of multimorbidity

**Fig 1:**
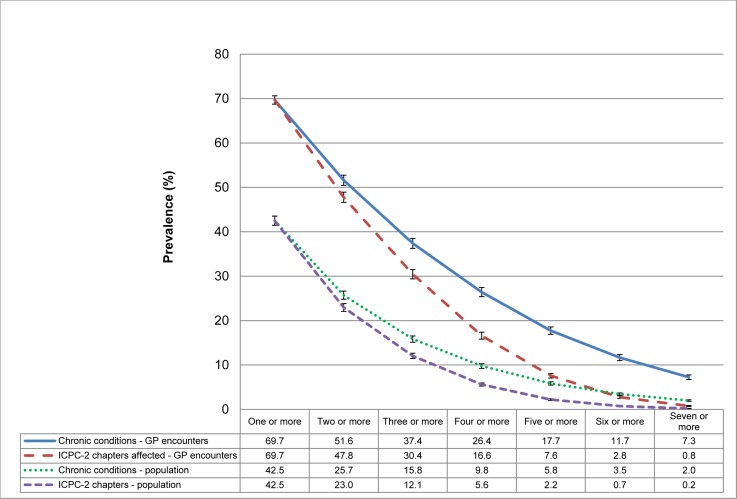
Prevalence of the number of chronic conditions among patients at encounters and people in the Australian population. (Note to go below Fig 1). Note: ICPC-2 chapters used as a proxy for body system.

About half (51.6%) the patients at GP encounters had two or more diagnosed chronic conditions and over one third (37.4%) had three or more. Nearly half (47.8%) had two or more body systems affected by chronic conditions and 30.4% had complex multimorbidity (Fig 1).

After adjustment we estimated that: 25.7% of the population had two or more diagnosed chronic conditions: 15.8% had three or more; 23.0% had two or more body systems affected by chronic conditions; and 12.1% of the population had complex multimorbidity (Fig 1).

## Discussion

Adjusting for each individual patient’s GP attendances over the previous 12 months provided prevalence estimates that were significantly lower than those generated by our previous method. This suggests that within an age-sex group, patients with diagnosed chronic conditions attend more often than those patients without. Adjusting for this variance will have made our population estimates more accurate than our previous estimates. We found that the clear majority of patients at GP encounters had at least one diagnosed chronic condition and about half had two or more. The most prevalent conditions among both patients at GP encounters and among people in the population were hypertension, osteoarthritis and hyperlipidaemia.

In our earlier prevalence papers we suggested that some of the differences between our prevalence estimates and those of the NHS may be due to respondent self-report error[4,5]. For instance the relatively high NHS prevalence estimates for rheumatoid arthritis may be due to respondents confusing it with ‘rheumatism’[5]. We found a similar difference in the current study. One of the great advantages of using the GP as an expert interviewer with access to the patient health record is that any such confusion from the patient can be clarified by the GP.

We estimated that about a quarter (25.7%) of the population had multimorbidity, two or more diagnosed chronic conditions, which is significantly smaller than the 32.6% estimated in our previous study(7). The lower estimates are due to using the new, more reliable method of estimating population prevalence of chronic conditions. However this revised estimate of the population prevalence multimorbidity remains significantly higher than the 23.0% estimated by the 2014–15 NHS[17]. This difference is probably due to the NHS only counting a selected list of chronic conditions while our study counted all chronic conditions. Previous research has shown that counting all chronic conditions provides the most reliable estimates of multimorbidity[27]. The issues of respondent accuracy noted above and the restricted list of chronic conditions used by the NHS, suggest that our estimates of multimorbidity may be more reliable than those of the NHS.

Our estimate of multimorbidity infers that 6.2 million patients would have been eligible to enrol in a Health Care Home' if eligibility was based on two or more diagnosed chronic conditions, as suggested from earlier Government statements. Our estimate of the proportion of patients at GP encounters with complex multimorbidity (30.4%) was higher than that found in our earlier study[7] (27.4%) and lower than that estimated by Brett et al (34.5%) among patients attending two GP practices in Perth[8].

Our study does have limitations. We have assumed that people who did not see a GP in the previous year, did not have a diagnosed chronic condition. This assumption may not hold for conditions such as mild asthma where a patient may not need to see a GP in a single chosen year. This may explain why our prevalence estimate for asthma was lower than that of the NHS.

The apparent over-representation of older patients attending a GP at least once in our study is probably due to comparing our sample to only the Medicare data. Medicare data would not include patients who only claimed DVA services that year. However, since patients who are covered by the DVA can also claim through Medicare, we could not combine those who made at least one claim in both datasets for fear of double counting the same patients. The DVA data is heavily skewed towards older patients (veterans of World War Two and their partners). It is likely that our estimated distribution of patients who attend general practice at least once in the previous year is actually far closer to reality than is implied by our comparison with Medicare claims data alone.

Our estimate of the average number of GP visits (4.54) for patients who had seen a GP at least once, was significantly lower than the average number of Medicare GP consultation items claimed per person, by those who claimed at least once (6.8 in 2014–15[31]). This means that the patients and GPs were under-reporting the number of GP visits made in the previous 12 months. This may be because the patient had seen another GP but had forgotten the visit(s) and/or did not wish the current GP to know of it. This under-reporting could have affected our national prevalence estimates if there was a bias for high or low attenders to under-report more often, and this cannot be assessed from the data.

## Conclusion

Of the three approaches we have tested to date, this study provides the most accurate method for estimation of population prevalence of chronic conditions using the GP as an expert interviewer, by adjusting for each patient’s reported attendance. The results provide the groundwork for the Australian Federal Government to cost and plan the rollout of the 'health care homes' initiative. If this initiative results in GPs enrolling high-need patients with multiple chronic conditions, the GPs will need to be properly compensated for switching from full fee-for-service to partial capitation. Further research is underway, examining the extent to which measures of multimorbidity can provide a structure for scientific calculation of appropriate capitation payments.

## Supporting information

S1 File Data points for Fig 1. (XLSX)

S2 File Example of BEACH recording form. (PDF)

S3 File Example of information form provided to GPs for this particular sub-study. (PDF)
