# Supplementary material for: The prevalence of diagnosed chronic conditions and multimorbidity in Australia: A method for estimating population prevalence from general practice patient encounter data
Source: PLoS One. 2017 Mar 9;12(3):e0172935. doi: 10.1371/journal.pone.0172935 (PMC5344344; doi:10.1371/journal.pone.0172935)
Supplement: S2 File — (PDF) [file pone.0172935.s002.pdf]

BEACH (Bettering the Evaluation And Care of Health) - Morbidity and Treatment Survey - National

© BEACH The University of Sydney 1996

DOC ID

Encounter Number

Date of encounter	Date of Birth	Sex	Patient Postcode
____/____/____	____/____/_ _ _ _	M ☐ F ☐	

	Yes / No	
New Patient	☐	☐
Health Care/Benefits Card	☐	☐
Veterans Affairs Card	☐	☐
NESB	☐	☐
Aboriginal	☐	☐
Torres Strait Islander	☐	☐

PATIENT SEEN BY GP ☐

PATIENT NOT SEEN BY GP ☐

Medicare Item Nos: (if applicable)

1. ____________
2. ____________
3. ____________

Home visit ☐

Workers comp paid ☐

Other paid ☐

No charge ☐

START Time

[:]

AM / PM (please circle)

Patient Reasons for Encounter

1.
2.
3.

Diagnosis/ Problem ① :

Problem Status New ☐ Old ☐ Work related ☐

Drug Name AND Form for this problem	Strength of product	Dose	Frequency	No. of Rpts	OTC	GP Supply	Drug status New	Drug status Cont.
1.								
2.								
3.								
4.								

Procedures, other treatments, counselling this consult for this problem

1. Prac Nurse? ☐ 2. Prac Nurse? ☐

Diagnosis/ Problem ② :

Problem Status New ☐ Old ☐ Work related ☐

Drug Name AND Form for this problem	Strength of product	Dose	Frequency	No. of Rpts	OTC	GP Supply	Drug status New	Drug status Cont.
1.								
2.								
3.								
4.								

Procedures, other treatments, counselling this consult for this problem

1. Prac Nurse? ☐ 2. Prac Nurse? ☐

Diagnosis/ Problem ③ :

Problem Status New ☐ Old ☐ Work related ☐

Drug Name AND Form for this problem	Strength of product	Dose	Frequency	No. of Rpts	OTC	GP Supply	Drug status New	Drug status Cont.
1.								
2.								
3.								
4.								

Procedures, other treatments, counselling this consult for this problem

1. Prac Nurse? ☐ 2. Prac Nurse? ☐

Diagnosis/ Problem ④ :

Problem Status New ☐ Old ☐ Work related ☐

Drug Name AND Form for this problem	Strength of product	Dose	Frequency	No. of Rpts	OTC	GP Supply	Drug status New	Drug status Cont.
1.								
2.								
3.								
4.								

Procedures, other treatments, counselling this consult for this problem

1. Prac Nurse? ☐ 2. Prac Nurse? ☐

NEW REFERRALS, ADMISSIONS Problem(s)

1. ______________ 1 2 3 4
2. ______________ 1 2 3 4

IMAGING/Other tests Body site Problem(s)

1. ________ - ________ 1 2 3 4
2. ________ - ________ 1 2 3 4
3. ________ - ________ 1 2 3 4

PATHOLOGY Problem(s)

1. ______________ 1 2 3 4
2. ______________ 1 2 3 4
3. ______________ 1 2 3 4

PATHOLOGY (cont) Problem(s)

4. ______________ 1 2 3 4
5. ______________ 1 2 3 4

Approx. how many times has this patient seen any GP in the past 12 months (including today)?

No: ________

☐ Don't know

Does the patient have any chronic conditions/ problems?

(Tick all that apply)

Musculoskeletal
- ☐ Osteoarthritis
- ☐ Rheumatoid arthritis
- ☐ Other arthritis
- ☐ Osteoporosis
- ☐ Chronic back pain

Psychological
- ☐ Depression
- ☐ Anxiety
- ☐ Insomnia
- ☐ Dementia (incl Alzheimer's)

Endocrine / nutritional
- ☐ Hyperlipidaemia
- ☐ Diabetes Type 1
- ☐ Diabetes Type 2
- ☐ Obesity (BMI ≥30)
- ☐ Hypothyroidism
- ☐ Hyperthyroidisim

Cardiovascular
- ☐ Hypertension
- ☐ IHD
- ☐ CHF
- ☐ Periph.Vasc. Dis
- ☐ CVA/stroke
- ☐ Atrial fibrillation

Other chronic problems
- ☐ Asthma
- ☐ COPD
- ☐ Sleep apnoea
- ☐ Chronic renal failure
- ☐ GORD
- ☐ Glaucoma
- ☐ Malignant neoplasm → Site: ____________

Other chronic problems not listed: *(please specify)*

____

____

____

☐ **NO chronic problems in this patient → Go to last question**

FINISH Time

[:]

AM / PM (please circle)

BL148B
