# Supplementary material for: The prevalence of diagnosed chronic conditions and multimorbidity in Australia: A method for estimating population prevalence from general practice patient encounter data
Source: PLoS One. 2017 Mar 9;12(3):e0172935. doi: 10.1371/journal.pone.0172935 (PMC5344344; doi:10.1371/journal.pone.0172935)
Supplement: S3 File — (PDF) [file pone.0172935.s003.pdf]

PLEASE READ CAREFULLY

The shaded section of the following forms asks questions about **PATIENT'S CHRONIC CONDITIONS / PROBLEMS.**

You may tear out this page as a guide to completing the following section of forms.

START Time

:

AM / PM
(please circle)

Start time

Record the time the consultation STARTED in hours and minutes and circle whether the time was AM or PM.

For example: 9 : 10

AM / PM
(please circle)

INSTRUCTIONS

Answer these questions for **EACH** of the **next 30 PATIENTS in the order in which the patients are seen.**

Please **DO NOT select patients** to suit the topic being investigated.

Use your own knowledge, patient knowledge and your records as you see fit, in order to answer these questions.

Finish time

Record the time the consultation FINISHED in hours and minutes and circle whether the time was AM or PM.

For example: 9 : 28

AM / PM
(please circle)

Frequency of GP visits

Please write the approximate **number of times (including today's visit)** the patient has **seen any GP for any reason** in the **past 12 months**. Use patient recall, and/or your notes or knowledge, to give the best estimate.

Abbreviations

BMI = body mass index
IHD = ischaemic heart disease
CHF = congestive heart failure
Periph Vasc Dis = peripheral vascular disease
CVA = cerebrovascular accident
COPD = chronic obstructive pulmonary disease (including emphysema)
GORD = gastro-oesophageal reflux disease

Patient chronic conditions/problems

The aim of these questions is to estimate the **prevalence** and **patterns** of **multimorbidity** in general practice patients. With an ageing population, the prevalence of multimorbidity is expected to increase and much of the care will fall on general practice. This study will highlight the complexity of multimorbidity and assist in planning for future health service needs.

If the patient has **NO chronic problems** please tick the box labelled '**NO chronic problems in this patient**', and go to the 'finish time' question.

If the patient **DOES** have **chronic conditions or problems**, please **use the tick boxes to indicate which ones** they have (irrespective of whether you have managed them today). Tick as many as apply.

If the patient has a **malignant neoplasm(s)** please **specify the primary site** of the neoplasm.

If the patient has any **other chronic problems or conditions** that are **not listed** please specify these in the **'Other chronic problems not listed'** section.

Approx. how many times has this patient seen any GP in the past 12 months (including today)?

No: ________

☐ Don't know

Does the patient have any chronic conditions/ problems?
(Tick all that apply)

Musculoskeletal
☐ Osteoarthritis
☐ Rheumatoid arthritis
☐ Other arthritis
☐ Osteoporosis
☐ Chronic back pain

Psychological
☐ Depression
☐ Anxiety
☐ Insomnia
☐ Dementia (incl Alzheimer's)

Endocrine / nutritional
☐ Hyperlipidaemia
☐ Diabetes Type 1
☐ Diabetes Type 2
☐ Obesity (BMI $\geq$30)
☐ Hypothyroidism
☐ Hyperthyroidisim

Cardiovascular
☐ Hypertension
☐ IHD
☐ CHF
☐ Periph.Vasc. Dis
☐ CVA/stroke
☐ Atrial fibrillation

Other chronic problems
☐ Asthma
☐ COPD
☐ Sleep apnoea
☐ Chronic renal failure
☐ GORD
☐ Glaucoma
☐ Malignant neoplasm→Site:________

Other chronic problems not listed: *(please specify)*
____
____
____

☐ **NO chronic problems in this patient→Go to last question**

FINISH Time

:

AM / PM
(please circle)

BL148B
